# Supplementary material for: A Bulk Oxygen Vacancy Dominating WO3−x Photocatalyst for Carbamazepine Degradation
Source: Nanomaterials (Basel). 2024 May 24;14(11):923. doi: 10.3390/nano14110923 (PMC11173414; doi:10.3390/nano14110923)
Supplement: Supplementary file 1 [file nanomaterials-14-00923-s001.zip › nanomaterials-2955351-supplementary.pdf]

Supporting information

A Bulk Oxygen Vacancy dominating WO_{3-x} Photocatalyst for Carbamazepine Degradation

Weiqing Guo [1], Qianhui Wei [1], Gangrong Li [1], Feng Wei [1,*] and Zhuofeng Hu [2,*]

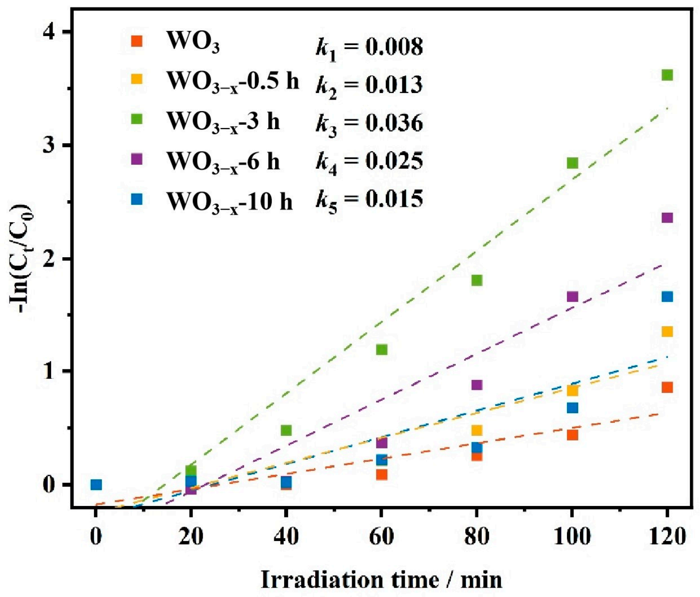

Figure S1. The pseudo first-order kinetic fitting curves of as-prepared samples.

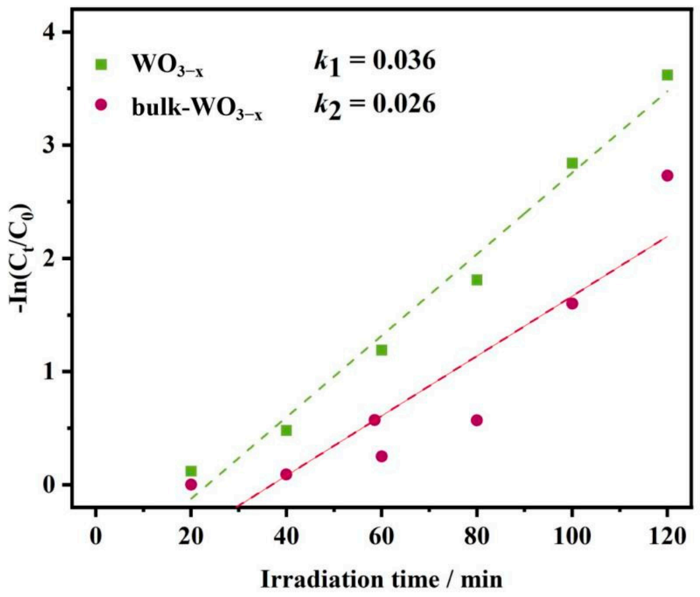

Figure S2. The pseudo first-order kinetic fitting curves of WO_{3-x} and bulk-WO_{3-x}.
